# Evidence That Two ATP-Dependent (Lon) Proteases in *Borrelia burgdorferi* Serve Different Functions

**DOI:** 10.1371/journal.ppat.1000676

**Published:** 2009-11-26

**Authors:** James L. Coleman, Laura I. Katona, Christopher Kuhlow, Alvaro Toledo, Nihal A. Okan, Rafal Tokarz, Jorge L. Benach

**Affiliations:** 1 State of New York Department of Health, Stony Brook University, Stony Brook, New York, United States of America; 2 Department of Molecular Genetics and Microbiology, Center for Infectious Diseases, Stony Brook University, Stony Brook, New York, United States of America; 3 Center for Infection and Immunity, Mailman School of Public Health of Columbia University, New York, New York, United States of America; Dartmouth Medical School, United States of America

## Abstract

The canonical ATP-dependent protease Lon participates in an assortment of biological processes in bacteria, including the catalysis of damaged or senescent proteins and short-lived regulatory proteins. *Borrelia* spirochetes are unusual in that they code for two putative ATP-dependent Lon homologs, Lon-1 and Lon-2. *Borrelia burgdorferi*, the etiologic agent of Lyme disease, is transmitted through the blood feeding of *Ixodes* ticks. Previous work in our laboratory reported that *B. burgdorferi lon-1* is upregulated transcriptionally by exposure to blood *in vitro*, while *lon-2* is not. Because blood induction of Lon-1 may be of importance in the regulation of virulence factors critical for spirochete transmission, the clarification of functional roles for these two proteases in *B. burgdorferi* was the object of this study. On the chromosome, *lon-2* is immediately downstream of ATP-dependent proteases *clpP* and *clpX*, an arrangement identical to that of *lon* of *Escherichia coli*. Phylogenetic analysis revealed that Lon-1 and Lon-2 cluster separately due to differences in the NH_2_-terminal substrate binding domains that may reflect differences in substrate specificity. Recombinant Lon-1 manifested properties of an ATP-dependent chaperone-protease *in vitro* but did not complement an *E. coli* Lon mutant, while Lon-2 corrected two characteristic Lon-mutant phenotypes. We conclude that *B. burgdorferi* Lons -1 and -2 have distinct functional roles. Lon-2 functions in a manner consistent with canonical Lon, engaged in cellular homeostasis. Lon-1, by virtue of its blood induction, and as a unique feature of the Borreliae, may be important in host adaptation from the arthropod to a warm-blooded host.

## Author Summary

Lyme disease, caused by the spirochete *Borrelia burgdorferi,* is the most prevalent arthropod-borne disease in North America. In nature, the bacterium oscillates between its tick vector host (*Ixodes* spp.) and small rodents (*Peromyscus* spp.). *B. burgdorferi* is able to persist in these two very different host environments by modulating the expression of surface lipoproteins proteins, or other proteins, in response to host factors or environmental cues such as temperature and pH. Our interest in this process led to the identification of a homolog of the *E. coli* ATP-dependent *lon* protease (*lon-1*) in *B. burgdorferi* that was upregulated in response to blood. The prototypical Lon of *E. coli* is a conserved protease important for the destruction of abnormal and short-lived proteins. *B. burgdorferi* is unusual in that it also codes for a second *lon* homolog, *lon-2*, that was not upregulated in response to blood. In this study, we sought to clarify the roles for Lon-1 and Lon-2 in *B. burgdorferi*. We present evidence that Lon-1 is an ATP- and Mg^2+^-dependent protease but does not function in a manner consistent with a prototypical Lon. Lon-2, however, functionally complemented Lon in *E. coli*. Thus, Lon-1 and Lon-2 appear to have distinct roles in *B. burgdorferi*; Lon-1 by virtue of its blood induction may be important in host adaptation, while Lon-2 is the functional homolog of *E. coli* Lon.

## Introduction

In nature, *Borrelia burgdorferi*, the agent of Lyme disease [1],[2], must adapt to disparate hosts, alternating between *Ixodes* ticks and various small rodent species. It is thought that this adaptation is made possible through the remodeling of the spirochete outer surface in response to environmental cues such as temperature [3],[4],[5], blood [6], pH [7],[8],[9],[10], and microbial density [11],[12],[13]. A recognized example of this is the reciprocal expression of outer surface lipoprotein (Osp) AB and OspC. OspA and OspB are dominantly expressed when the spirochete is in culture or in the midgut of a flat unfed tick, then downregulated upon feeding and subsequent exposure to blood, increased temperature, and a drop in pH. OspC is concomitantly upregulated [3],[14],[15],[16]. At this time the spirochetes experience a period of vigorous growth and migrate from the tick midgut to the salivary glands via the hemolymph [17]. This is followed by transmission to the mammalian host. The coordinated expression of OspAB and OspC has been proposed as an example of spirochete-vector interaction, with OspAB and OspC being implicated in spirochete adhesion to the tick midgut [18],[19],[20] and salivary gland respectively. At the time of feeding, abundant, surface exposed OspA and OspB need to be broken down to remodel the outer membrane. We have had a long-standing interest in the proteases of *B. burdorferi* and their functions. In the absence of known secreted proteases in the genome of *B. burgdorferi* [21], we have documented the reliance of this organism on borrowed proteolytic activity. The plasminogen activation system is used by the spirochete to cross cellular and extracellular matrices by inducing the production of and/or incorporating enzymatically active plasmin, urokinase plasminogen activator, and metalloproteases onto its surface [15],[22],[23],[24],[25] in both vectors and vertebrate hosts. While plasmin was important in promoting the migration of *B. burgdorferi* through the tick, this mammalian protease did not have an effect on the remodeling the outer surface of the spirochete at this crucial time. However, OspC, which is the upregulated lipoprotein during tick feeding is a plasminogen receptor [26] providing further indication that this system is associated with migration of the spirochete in the vector.

In a previous study, our interest in the proteolytic remodeling of outer surface lipoproteins during the transition of the spirochete from tick to mammalian host led us to examine the *B. burgdorferi* transcriptome after exposure *in vitro* to increased temperature in the presence and absence of blood [6]. The changes that we observed for the blood condition included the upregulation of OspC, other lipoproteins, and many genes of known or unknown function. One intriguing observation was the significant upregulation of a putative ATP-dependent protease La (Lon-1, BB0253) [21], a homolog of the *Escherichia coli lon* gene [27] and identical to the *lon* gene described for *B. burgdorferi* by Cloud et al. [28]. The *B. burgdorferi* genome also codes for a second putative ATP-dependent *lon* homolog, BB0613 (*lon-2*) [21], which was not differentially expressed in the array [6].

The canonical *E. coli* Lon (Lon-Ec), a conserved and much studied protease important for intracellular degradation of short-lived and abnormal proteins [27], has also been identified as a DNA-binding protein [29], and as a part of the heat shock regulon [30],[31]. Besides bacteria, Lon homologs have also been found in other organisms including archaea [32], yeast [33],[34],[35], and animals [36],[37]. It is an 87-kDa oligomeric serine protease with a structure consisting of three functional domains: the heterogeneous NH_2_-terminal (LAN) domain, the ATPase domain, and the C-terminal proteolytic domain. Structural studies have demonstrated that Lon is a ring-shaped complex composed of multiple identical 87-kDa subunits, with Lon-Ec functioning as a hexamer [38]. Lon plays an important role in homeostasis by targeting abnormal proteins and unstable regulatory proteins such as the SulA division regulator [39] and RcsA, the positive regulator of capsular polysaccharide [40],[41], and degrading tmRNA-tagged proteins during *trans* translation [42]. In addition, Lon has been shown to be a virulence factor for infection in mice [43],[44]. Our interest in *B. burgdorferi* Lon-1 stemmed from the idea that as a protease, it could take part in the remodeling of the spirochete outer surface during the period following a blood meal in the tick. With this in mind, we sought to identify functional roles for Lon-1 and Lon-2 through phylogenetic analysis, and biochemical and genetic means. Our results support the idea that Lon-2 fulfills the role of canonical Lon and is involved in cellular quality control and homeostasis, while Lon-1 plays a role in host adaptation during the transition from tick to mammal.

## Results/Discussion

### Spirochetal Lon-1 and Lon-2 are only distantly related to canonical Lon

**Figure 1:**
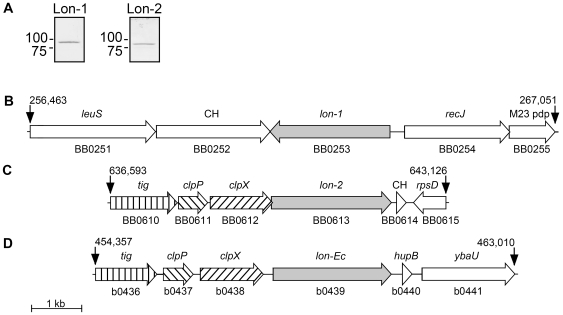
Native Lon expression in *B. burgdorferi* and comparison of *lon* loci and flanking genes from *B. burgdorferi* and *E. coli*. (A) Native Lon-1 and Lon-2 expression in *B. burgdorferi* wild-type BB050.14 spirochetes. Lon-1 and Lon-2 were detected in whole cell lysates by use of rabbit and mouse antiserum, respectively. (B) *B. burgdorferi lon-1* region. (C) *B. burgdorferi lon-2* region. (D) *E. coli lon* region. Gene symbols and gene loci (The Institute for Genomic Research-TIGR) are given above and below each gene, respectively. CH, conserved hypothetical; *leuS*, leucyl-tRNA synthase; M23 pdp, M23 peptidase domain protein; *tig*, trigger factor; *clpP*, ATP-dependent proteolytic subunit of ClpA-ClpP serine protease; *clpX*, ATP-dependent specificity component of ClpP serine protease; *rpsD*, ribosomal protein S4; *hupB*, DNA-binding protein HU-beta; *ybaU*, putative protease maturation protein. Gene coordinates are indicated with arrows. One-kb size marker is shown at lower left.

*B. burgdorferi* codes for two putative ATP-dependent Lon homologs designated Lon-1 and Lon-2 [21]. In a previous study, Lon-1 was upregulated in response to blood, while Lon-2 was not [6], though each is expressed by *B. burgdorferi* during culture in BSK-H medium (Figure 1A). The presence of more than one *lon* gene homolog is rare in the eubacteriae, but appears to be a unique characteristic of the genus *Borrelia*, as the genomes for *B. afzelii* [45], *B. garinii* [46], *B. recurrentis* [47], *B. duttoni* [47], and *B. hermsii* [48] also code for two Lon homologs. Our interest in Lon-1 stemmed from its hypothetical function as a protease, and we hypothesized that it could be involved in the remodeling of spirochete outer surface lipoproteins during the transition from the tick to the mammal, thus warranting its investigation as a potential virulence factor. By extension, our hypothesis would imply that Lon-2 would likely function in a manner similar to that of Lon-Ec. The canonical *Lon-Ec* resides on the chromosome, adjacent to and downstream from two other ATP-dependent proteases, *clpP* and *clpX*, the proteolytic subunits comprising the ATP-dependent *clpXP* complex thought to share its role in cellular housekeeping. Examination of the chromosomal location of *B. burgdorferi lon*-2 reveals that it is arranged identically, immediately downstream of *clpX* and *clpP*, while Lon-1 is on a distant part of the chromosome (Figure 1). The *lon-2* arrangement shown for *B. burgdorferi* in Figure 1 is maintained in other *Borrelia*, while *Treponema* and *Leptospira* code for single *lon* genes that are not arranged in the same fashion as canonical *lon* (data not shown).

**Figure 2:**
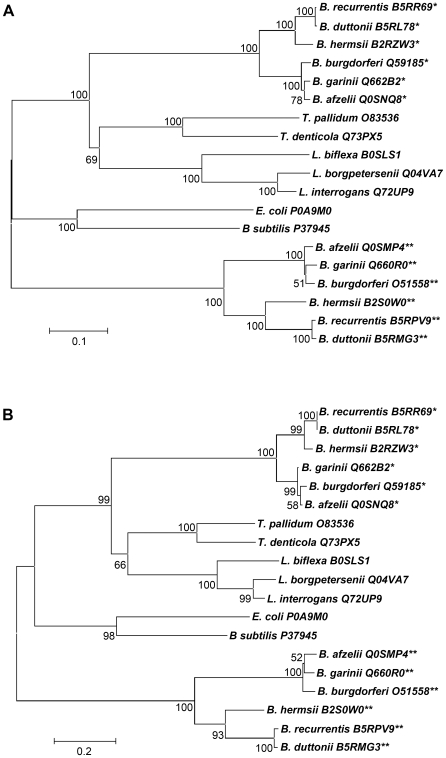
Phylogenetic trees based on Lon amino acid sequences of spirochetes. (A) Phylogenetic tree of spirochetes derived from the amino acid sequences of Lons from reference strains of *B. burgdorferi* sensu lato and relapsing fever *Borrelia*, *Leptospira* and *Treponema* species with *E. coli* and *B. subtilis* as an external group. (B) Phylogenetic tree of spirochetes derived from the amino acid sequences of the amino-terminal NH_2_-terminal domains from reference strains of *B. burgdorferi* sensu lato and relapsing fever *Borrelia*, *Leptospira* and *Treponema* species, with *E. coli* and *B. subtilis* as outgroups. The neighbour joining trees were constructed with MEGA3 software. Bootstrap confidence levels above 50% are indicated to left of each relevant cluster. Asterisks: * Lon-1; ** Lon-2. All of the sequences were obtained from Swiss-Prot Protein knowledgebase. Accession numbers are listed next to each species. Distance marker is shown at lower left for each panel.

Because the similarity of the chromosomal location of Lon-2 to canonical *E. coli lon* suggested that they could serve a similar function, we tested the premise that Lon-1 of *B. burgdorferi* may have other functions that could be unique to spirochetes, as transcription of this gene was upregulated on blood induction. Our assessment of the premise began with comparison of amino acid sequences (defined by Pfam, PF00004, PF02190, and PF05362) of *Borrelia* Lons with the Lon sequences of other spirochetes and more distantly related bacteria through the construction of phylogenetic trees (Figure 2). The trees we created included Lon amino acid sequences from spirochetes coding for two Lons (Lyme disease and relapsing fever Borreliae) and single Lons (*Treponema*, *Leptospira*, *E. coli*, and *B. subtilis*). In Figure 2A, a tree based on the entire amino acid sequences of Lon proteins is shown. One evident feature from the tree is that while all of the Lons are related to some extent, they began to diverge in the distant past. The *Borrelia* Lon-1s cluster together, indicating that they are closely related. Within the Lon-1 group, the relapsing fever Borreliae, consisting of *B. recurrentis*, *B. duttoni*, and *B. hermsii* form a subcluster, while the Lyme disease group, *B. burgdorferi*, *B. afzelii*, and *B. garinii* form another subcluster. Although distinct, *Borrelia* Lon-1 is also related to Lons of *Treponema* and *Leptospira*, suggesting a common ancestor. Lon-2 sequences from Lyme disease and relapsing fever Borreliae cluster separately from all the other bacteria, indicating a close relationship exclusive to the genus *Borrelia*, and form subclusters identical to those of Lon-1. We also performed the phylogenetic analyses using *lon* nucleotide sequences, with virtually identical results (data not shown).

The carboxy-terminal proteolytic domain and the ATPase domain of Lon are conserved across different bacterial species, whereas the NH_2_-terminal domain, which is responsible for substrate binding, is heterogeneous, and thus, the most useful for phylogenetic analysis. To determine a possible functional difference between Lon-1 and Lon-2, we tested the phylogenetic relatedness of the NH_2_-terminal domains of the bacteria analyzed in Figure 2A (Figure 2B). Overall, the clustering pattern for the NH_2_-terminal domain sequences closely resembles that of the whole molecule, implying that the clustering is driven by differences in the NH_2_-terminal domain alone. Perhaps the most significant outcome of the phylogenetic analysis is that the NH_2_-terminal domains of Lon-1 and Lon-2 are shown to be especially dissimilar, which may correlate with substrate specificity. Thus, Lon-1 and Lon-2 may each recognize different substrates. Another significant outcome of the phylogenetic analysis is that neither Lon-1 nor Lon-2 are closely related to the canonical Lons of *E. coli* and *B. subtilis*, preventing us from making sequence-driven inferences about the function of either *Borrelia* Lon.

### Recombinant Lon-1 is enzymatically active

**Figure 3:**
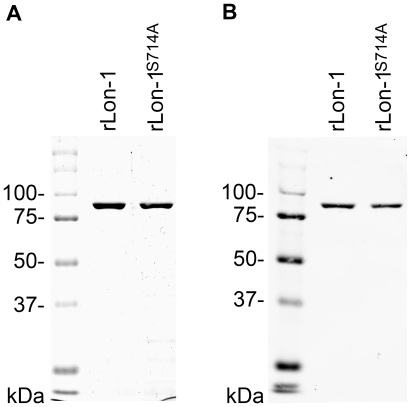
SDS-PAGE and western blot analysis of purified recombinant wild-type and mutant (S714A) *B. burgdorferi* Lon-1 protein. (A) Coomassie blue-stained 12.5% gel of recombinant Lon-1 wild-type (rLon-1) and S714A mutant (rLon-1^S714A^). (B) Corresponding western blot using rabbit polyclonal antiserum.

To further examine *B. burgdorferi* Lon function, we characterized the Lon-1 gene product to study its structure and test it for biological activity. For this purpose, we generated wild-type rLon-1 (strains and PCR primers are given in Tables 1 and 2). Proteolytic domains of Lons typically contain a lysine-serine dyad critical for catalysis with the lysine appearing 43 residues downstream of the catalytic serine [38]. Inspection of the deduced Lon-1 amino acid sequence shows that it contains a serine residue a position 714 (Swiss-Prot predicted active site S714) and a lysine at position 757 (Swiss-Prot predicted active site K757), therefore, we also generated a recombinant with the putative catalytic serine714 mutated to alanine (S714A) to abolish proteolytic activity and confirm its identity as part of the catalytic dyad. Recombinant wild-type (rLon-1) and mutant Lon-1 (rLon-1^S714A^) was analyzed by SDS-PAGE and western blot (Figure 3) to assess their purity and confirm their size. Figure 3 panels A and B show that rLon-1 and rLon-1^S714A^ were purified to homogeneity and their SDS-PAGE profiles are consistent with their predicted size (native Lon-1 has a predicted MW of 90,701 and a predicted MW of 92,864 with the attached His-tag).

**Table 1:** Bacterial strains and plasmids used in this study.

	Strain or plasmid name	Genotype or description	Reference or source
***B. burgdorferi***	B31MI	MedImmune strain, wild-type	Reference [21]
	B31A3	cp9 , – wild-type	Reference [74]
***E. coli***	BL21 Star™(DE3)pLysS	Expression host, F– ompT hsdSB (rB–, mB–) gal dcm rne131 (DE3) pLysS (CamR)	Invitrogen, Carlsbad, CA
	Rosetta™ 2(DE3)pLysS	Expression host, F- ompT hsdSB (rB- mB-) gal dcm (DE3) pLysS RARE2 (CamR)	Novagen, Gibbstown, NJ
	HDB97	SG22622 *mal+*, wild-type *lon*	Reference [68]
	HDB98	HDB97 (*lon^510^*)	Reference [68]
	HDB98pBAD24	HDB98 containing pBAD24	This study
	HDB98pBAD*lon-1*	HDB98 containing pBAD24*lon-1*	This study
	HDB98pBAD*lon-2*	HDB98 containing pBAD24*lon-2*	This study
***Plasmids***	pET30a(+)	Expression vector	Novagen
	pCK003.9	pET30a(+) expressing wt rLon-1	This study
	pCK004.3	pET30a(+) expressing rLon-1^S714A^	This study
	pCK006.2	pET30a(+) expressing wt rLon-2	This study
	pBAD24	Expression vector containing P_BAD_	Reference [75]
	pBAD*lon-1*	pBAD24 with *lon-1* cloned in	This study
	pBAD*lon-2*	pBAD24 with *lon-2* cloned in	This study

**Table 2:** Oligonucleotide primers used in this study.

Primer Pair	RS^a^	Primer sequence (5′→3′)	Application
P1F P2R	NdeI XhoI	TTACATATGGATGAATCTAAAAAAGCTAGG TTACTCGAGAAACAATAATTTAATGACCTCG	Amplification of *lon-1* for cloning into pET30a
P21F P22R	NdeI XhoI	CTCCCATATGAAATCAATCTTAAATATGAAACCCTCGAGAATAATATTCAAATAATCAAAAACTTC	Amplification of *lon-2* for cloning into pET30a
P3F P4R	— —	CCCAAAAGATGGGCCTGCTGCAGGAATTAC AGCTATTGTAATTCCTGCAGCAGGCCCATC	Amplification of *lon-1* for site-directed mutagenesis
P23F P24R	— —	CCAAAAGATGGGCCTGCTGCAGGTATTACCATTGCAAC GTTGCAATGGTAATACCTGCAGCAGGCCCATCTTTTGG	Amplification of *lon-2* for site-directed mutagenesis
P50F P51R	NcoI NcoI	CGCGAGCCATGGATGAATCTAAAAAAGCTAGG CGCGAGCCATGGTTAAAACAATAATTTAATGACC	Amplification of *lon-1* for cloning into pBAD24
P52F P53R	NheI KpnI	CGCGAGGCTAGCAGGAGGAATTCACCATGATAAAAAATAGAAAAGAAG CGCGAGGGTACCTTAAATAATATTCAAATAATC	Amplification of *lon-2* for cloning into pBAD24

All PCR primers shown here were designed for this study.

a Restriction sites are underlined.

**Figure 4:**
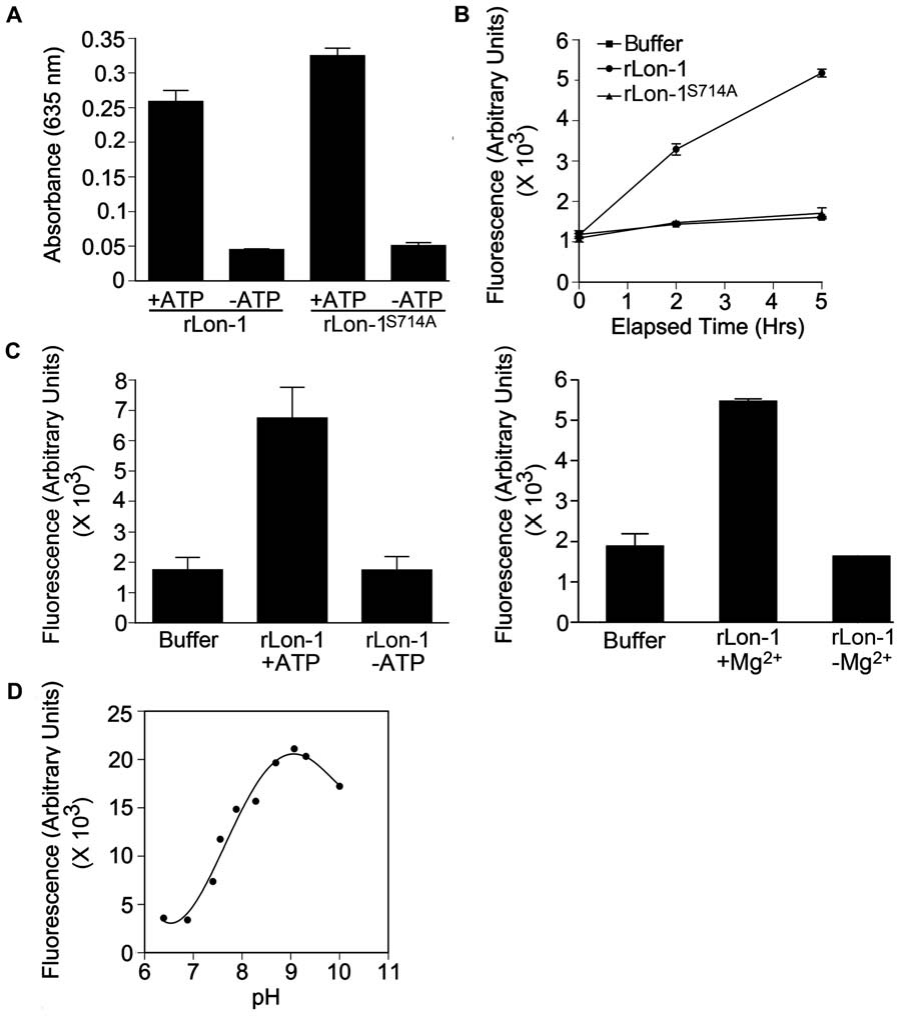
Enzymatic activities of purified recombinant wild-type and mutant Lon-1. (A) Colorimetric determination of hydrolysis of ATP by wild-type (rLon-1) and mutant (rLon-1^S714A^) Lon-1, with or without ATP substrate. (B) Time course of proteolysis of the substrate FITC-casein by rLon-1 and rLon-1^S714A^. (C) ATP (left panel) and Mg^2+^ (right panel) requirements for proteolysis of FITC-casein by rLon-1. (D) Dependence on pH for proteolysis of FITC-casein by rLon-1. Experiments shown here are representative of two or more experiments in which similar results were obtained. Error bars represent the standard deviation of duplicate samples.

To examine the catalytic activities of wild-type rLon-1 and rLon-1^S714A^ we conducted ATPase and caseinolytic assays (Figure 4). Analysis of ATPase activity by a colorimetric dye-binding assay showed that both the rLon-1 and the rLon-1^S714A^ exhibited similar levels of activity (Figure 4A). The ATPase activity of rLon-1^S714A^ confirmed that the ATPase and the proteolytic domains function independently and that introduction of a mutation into the proteolytic domain does not affect the ability of Lon-1 to hydrolyze ATP. To test for rLon-1 and rLon-1^S714A^ ATP-dependent proteolytic activity we measured the degradation of α-casein-fluorescein isothiocyanate (FITC-casein), in the presence of 10 mM ATP and 4 mM ATP [33],[37],[49]. In contrast to the ATPase assay, the rLon-1^S714A^ did not show activity levels above that of the buffer control while the wild-type rLon-1 degraded FITC-casein in a time-dependent manner (Figure 4B). This confirms that through targeted mutagenesis of serine714, the catalytic serine residue was identified correctly. Omission of ATP from the assay verified that it is required for the degradation of FITC-casein by rLon-1, as would be expected for an ATP-dependent protease (Figure 4C, left panel).

The presence of Mg^2+^ is critical for assembly of both *E. coli* and *Mycobacterium smegmatis* Lon subunits into proteolytic multimeric structures [50],[51]. To investigate whether the caseinolytic activity of Lon-1 was Mg^2+^-dependent we carried out the caseinolytic assay with wild-type rLon-1 in the presence and absence of Mg^2+^ (for the absence of Mg condition, MgCl_2_ was omitted from the reaction buffer). In the presence of Mg^2+^ the catalytic activity was retained, however, the absence of Mg^2+^ reduced the activity to background levels (Figure 4C, right panel). This result implies that the assembly of Lon-1 monomers into multimeric structures is essential for proteolytic activity. We also examined the caseinolytic activity of wild-type rLon-1 over a gradient of pH conditions. The pH optimum for wild-type rLon-1 ranged from 9.0 to 9.5, which is similar to that of Lon-Ec [52] (Figure 4D). Exhaustive attempts to overexpress rLon-2 proved unsuccessful, as the detergent-soluble product was proteolytically inactive, precluding further use.

### Lon-1 does not degrade Borrelia-SsrA tagged reporter protein *in vitro*

**Figure 5:**
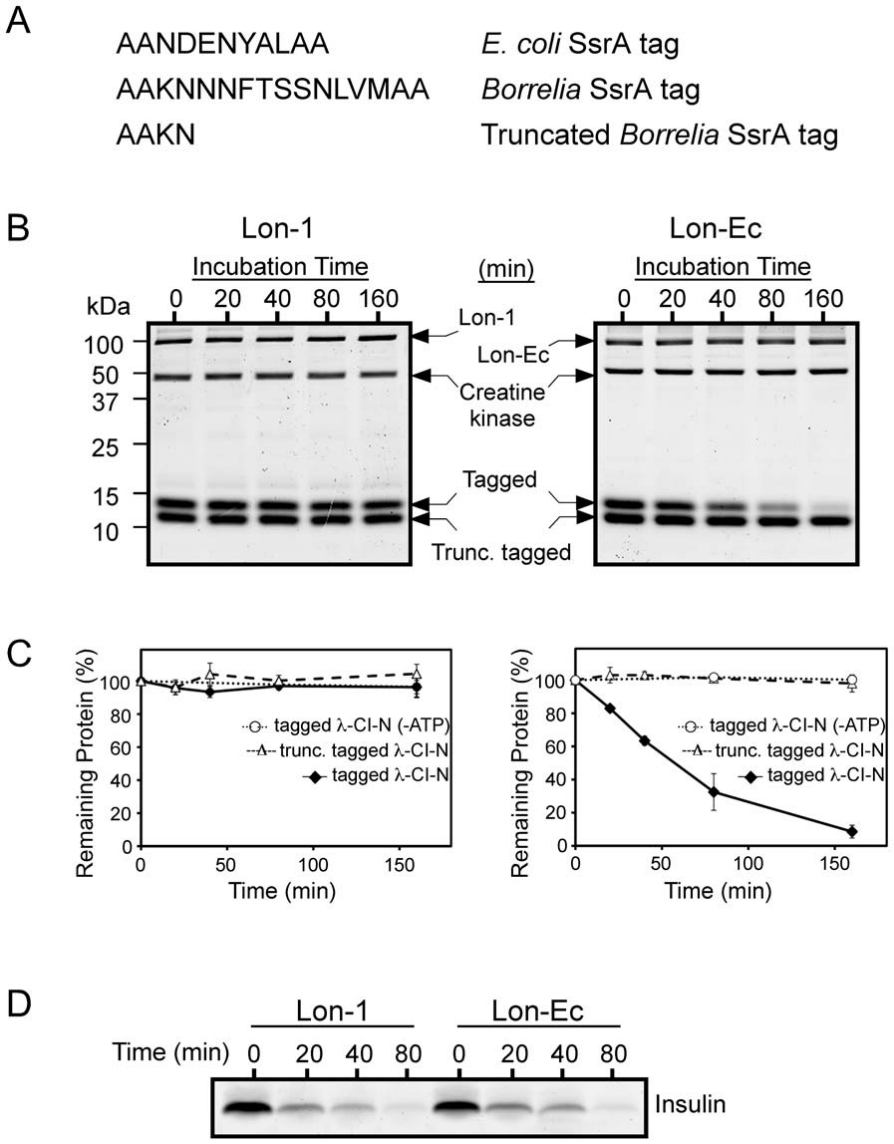
Lon-1 does not degrade λ-CI-N reporter protein carrying a *Borrelia*-SsrA tag. (A) SsrA-encoded proteolysis tags are shown. Truncated *Borrelia* SsrA tag refers to the reporter protein carrying a partial SsrA tag (determined by mass spectroscopy). (B) *In vitro* proteolysis assay was performed as described and protein samples taken at indicated time points were resolved on 15% polyacrylamide Tris-tricine gel followed by Coomassie blue staining. Protein bands labeled as tagged and trunc. tagged refer to the λ-CI-N reporter protein carrying a full-length or truncated *Borrelia* SsrA tag, respectively. Creatine kinase, present in the reaction mixture, was used as a loading control. (C) Protein gels were quantitatively analyzed using the Odyssey infrared imaging system (LI-COR) and remaining reporter proteins were calculated and presented as a percentage (average of three independent experiments). Error bars represent standard deviations. (D) Proteolysis assay of insulin was performed in a manner similar to λ-CI-N reporter *in vitro* assays.

A variety of *in vivo* Lon substrates have been identified to date, including SulA, RcsA, and UmuD [40],[53],[54],[55]. In addition, rLon-Ec was reported to degrade SsrA-tagged proteins, a characteristic that could be directly examined *in vitro* [42]. SsrA (also known as tmRNA) is a unique RNA molecule that is highly conserved in *Eubacteria* and plays a major role in dealing with stalled ribosomes caused by aberrant mRNAs. In this process, SsrA co-translationally places a peptide tag on the carboxyl termini of the incomplete polypeptides. These marked polypeptides are recognized by cellular proteases, including Lon, and readily degraded [56]. The *ssrA* sequence of *B. burgdorferi* has been identified and the presence of SsrA RNA product has been confirmed [57]. To test whether Lon-1 can degrade proteins marked with a *Borrelia*-SsrA tag, we constructed and purified a reporter protein, λ-CI-N-SsrA^Bb^, which carries a *Borrelia*-SsrA tag on the C-terminus. When purified product was analyzed, we observed two distinct protein bands of similar sizes, most likely artifacts of the purification procedure (Figure 5B, low MW proteins). Analysis of each protein by MALDI-TOF mass spectroscopy showed that the higher molecular weight protein is λ-CI-N-SsrA^Bb^ containing a full-length SsrA tag, and the lower molecular weight is the same protein with a truncated SsrA tag (Figure 5A). Next, we performed *in vitro* degradation assays using purified rLon-1 or rLon-Ec and the reporter protein to directly evaluate Lon activity. Assays showed that rLon-1 degraded neither tagged λ-CI-N reporter nor truncated tagged λ-CI-N reporter proteins (Figure 5B,C). Interestingly, rLon-Ec could effectively degrade tagged λ-CI-N reporter despite a significant amino acid sequence difference between *Borrelia* and *E. coli* SsrA tags. Truncated SsrA tagged reporter protein was not degraded by rLon-Ec, suggesting that it specifically recognized the SsrA tag, not the λ-CI-N protein itself. Furthermore, rLon-Ec required ATP for the degradation of the λ-CI-N-SsrA^Bb^ reporter protein (Figure 5C).

To further analyze the protease activity of Lon *in vitro*, we used insulin as a substrate in an *in vitro* assay similar to that of the tagged λ-CI-N reporter. Assays showed that both Lon-1 and Lon-Ec could effectively degrade insulin (Figure 5D). These results suggest that unlike Lon-Ec, Lon-1 of *B. burgdorferi* is an active protease that does not recognize *Borrelia*-SsrA tagged proteins *in vitro*.

### Mutant Lon-1 inhibits the aggregation of insulin β-chain

**Figure 6:**
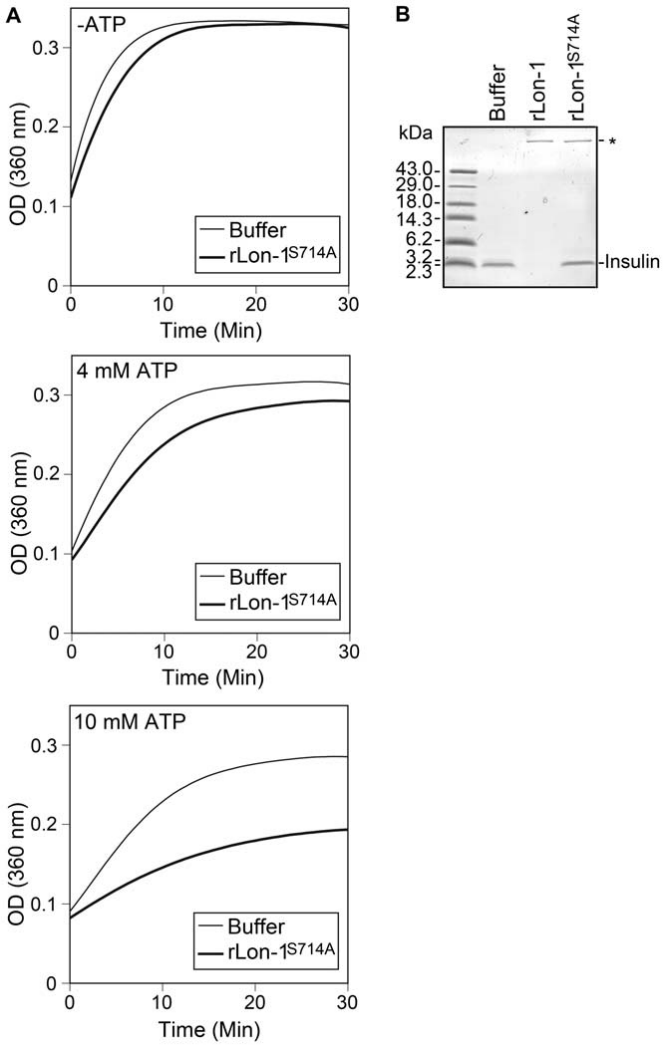
Chaperone-like activity of rLon-1^S714A^, as determined by prevention of the aggregation of reduced insulin B-chain. (A) Effect of rLon-1^S714A^ on aggregation of reduced insulin B-chain in the absence of ATP (upper panel), in the presence of 4 mM ATP (middle panel), and in the presence of 10 mM ATP (lower panel). (B) Effect of wild-type rLon-1 and rLon-1^S714A^ on reduced insulin as analyzed by Tris-tricine SDS-PAGE, followed by Coomassie blue staining. For the digestion, approximately 25 µg of rLon-1 and rLon-1^S714A^ were incubated at 37°C for two hours with 150 µg of insulin, in the presence of 4 mM ATP. Each gel lane received 10 µl of the digestion mixture. Asterisk shows position of either rLon-1 or rLon-1^S714A^. Experiments shown are representative of two experiments in which similar results were obtained.

In addition to its function as an ATP-dependent protease, Lon exhibits a chaperone-like role in mammalian mitochondria, yeast, and bacteria [58],[59],[60],[61],[62]. Cloud et al. [28] reported that complementation of an *E. coli lpxA* temperature-sensitive mutant with sequence corresponding to the N-terminus and ATP-binding site of *B. burgdorferi lon-1* allowed the mutant to grow at the non-permissive temperature. One reason for the suppression of the temperature-sensitive phenotype could be a novel chaperone-like activity conferred to *lon-1* through the absence of the proteolytic domain from the construct, thus allowing stabilization of the mutant *lpxA* gene. To further characterize Lon-1 we investigated its capacity to act as a chaperone by use of an *in vitro* assay employing bovine insulin. Insulin is a 6-kDa polypeptide consisting of two chains, A and B, connected by two disulfide bridges. Reduction of the molecule results in aggregation of the B-chain, while the A-chain remains soluble [63]. The chaperone assay is based upon the prevention of B-chain aggregation through interaction with an added chaperone. Assay results are measured via light scattering caused by insulin aggregates [59],[61]. Therefore, to determine if rLon-1 possessed chaperone-like activity, we tested the ability of rLon1 and rLon-1^S714A^ to inhibit the aggregation of insulin B-chain under reducing conditions. Kinetic spectrophotometric analysis revealed that in the absence of added Lon protein (buffer only), an irreversible formation of B-chain aggregates was the result. Addition of rLon-1^S714A^ without ATP did not significantly affect aggregate formation (Figure 6A, top panel). However, addition of rLon-1^S714A^ in the presence of increasing amounts of ATP resulted in a dose-dependent inhibition of aggregate formation (Figure 6A, lower panels). Wild-type rLon-1 enzymatically degraded the insulin and thus was not suitable for use in the assay (Figure 6B).

The preservation of chaperone-like activity despite mutagenesis of the proteolytic site in Lon-1 is consistent with that found in yeast and mammalian Lon [58],[60]. The critical requirement for ATP in our Lon-1 chaperone assay results is in agreement with that reported for chaperones in general [64] and for yeast Lon [58],[60], but differs with one study in which the chaperone-like activity of a bacterial Lon was investigated. In that report, Lon from *Brevibacillus thermoruber* prevented insulin aggregation in an ATP-independent manner [59].

Protein stabilization and catalysis would seem to be opposing actions; nevertheless, ATP-dependent chaperone-proteases of the Clp and Lon families are well documented [62]. In the case of Lon-1, the demonstration of a chaperone-like activity *in vitro*, while interesting, does not definitively establish a role as a chaperone *in vivo*. On the other hand, differential recognition of potential substrates λ-CI-N-SsrA^Bb^ and insulin or casein by rLon-1 demonstrates an ability by the enzyme to discriminate, implying that it could act as a chaperone for one protein and degrade another.

### Lon-2 complements an *E. coli* lon mutant

The implied redundancy of retaining two Lon genes necessarily raises the question of whether they are in actuality functionally equivalent, complementary, distinct, or overlapping, which may be reflected by similarities or differences in composition or expression pattern. *B. burgdorferi* Lons-1 and -2 are comparable in size (∼90 kDa) and domain structure. However, Lon-2 may be more chemically similar to the archetypal Lon-Ec, as suggested by the similarity of their isoelectric points (Lon-2: pI = 6.04/Lon-Ec: pI = 6.32), and the similarity in its chromosomal arrangement to Lon-Ec. Lon-1, on the other hand, is an exceedingly basic protein (pI = 9.85). In our previous report, *lon-1* was transcriptionally upregulated *in vitro* when exposed to blood and temperature shift in comparison to temperature shift alone. *Lon-1* upregulation in response to temperature shift alone was not determined in this study [6]. However, in other studies, *lon-1* was not activated by temperature [65],[66], pH shift [66], or by cultivation in dialysis membrane chambers in rats [66],[67]. In line with our own findings, none of the above microarray studies reported the differential expression of *lon-2* as a result of any condition [65],[66],[67].

**Figure 7:**
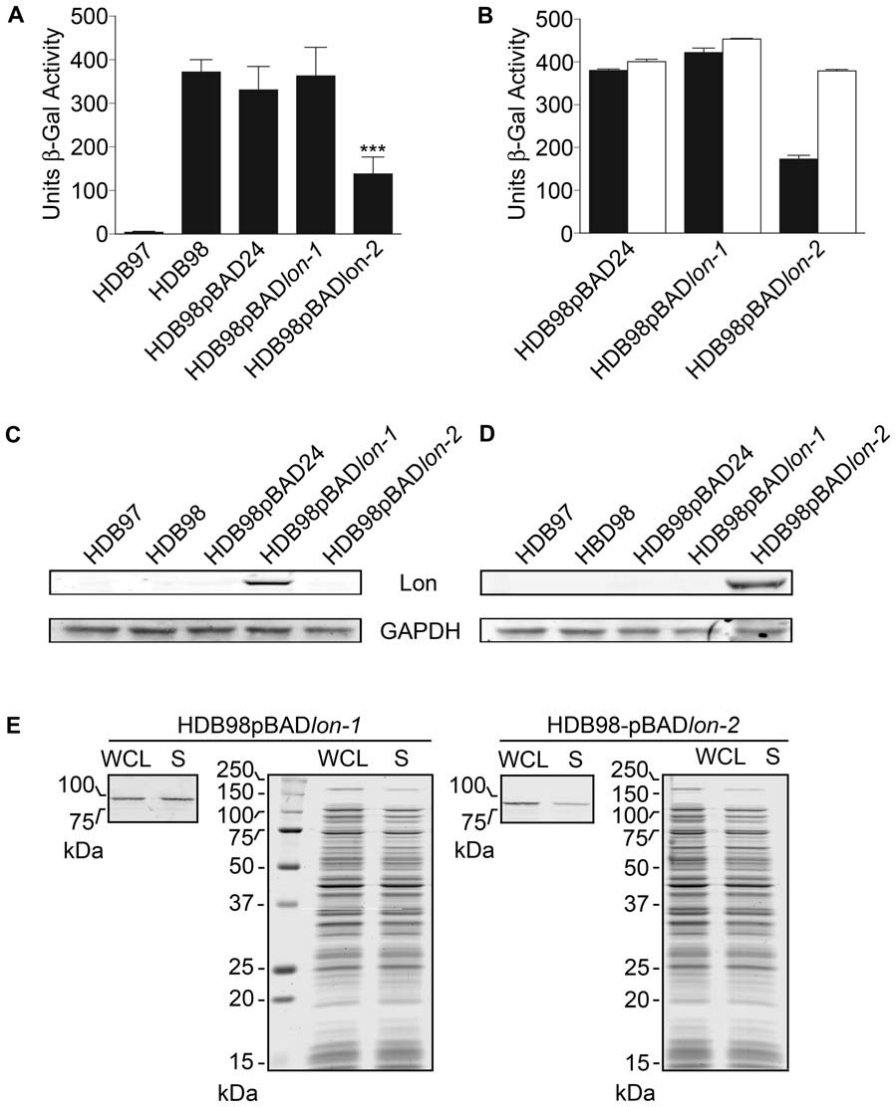
*lon-2*, but not *lon-1*, complements an *E. coli lon* mutant. (A) β-galactosidase activity of wild-type HDB97 (*cpsB*::*lacZ*), *lon* mutant HDB98 (HDB97*lon* ^510^), and HDB98 transformed with empty pBAD24 vector (HDB98pBAD24) or vector containing *B. burgdorferi lon-1* (HDB98pBAD*lon-1*) or *lon-2* (HDB98pBAD*lon-2*). Figure represents the combined data from two independent experiments. HDB97 and HDB98 were cultured in LB medium and HDB98pBAD24, HDB98pBAD*lon-1*, and HDBpBAD*lon-2* were cultured in LB medium supplemented with 0.1% arabinose. ***, significantly different from HDB98, HDB98pBAD24, and HDB98pBAD*lon-1* (P<0.001, Tukey-Kramer Multiple Comparisons Test). Error bars represent standard deviations. (B) β-galactosidase activity of *E. coli* grown with (black bars) and without (white bars) 0.1% arabinose. Figure is representative of two independent experiments that produced similar results. Error bars in panels A and B represent the standard deviations of triplicate samples. (C, D) Western blot analysis of culture whole cell lysates from experiment depicted in panel A showing Lon-1 and Lon-2 expression. Glyceraldehyde-3-phosphate dehydrogenase (GAPDH) was used as a loading control. (E) Solubility of Lon-1 and Lon-2 protein produced by HDB98pBADlon-1 and HBD98pBADlon-2, respectively. WCL, whole-cell lysate; S, soluble fraction (supernatant) after centrifugation for 10 minutes at 16,000× g. For each *E. coli* strain, western blots are shown on the left. Corresponding Coomassie Blue-stained protein is shown on the right to verify that equivalent amounts of protein were loaded.

We further investigated the respective roles of *lon-1* and *lon-2* by testing experimentally whether either gene is functionally equivalent to *lon-Ec*. Use of a complementation strategy allowed us to investigate and compare the role(s) of *lon-1* and *lon-2* in an *in vivo* context and circumvent the inability to produce rLon-2. Lon-Ec specifically degrades RcsA, a positive activator for transcription of capsular polysaccharide (*cps*) genes [40],[41]. Lon-deficiency in *E. coli* causes an accumulation of *RcsA* and a concommitant excess of CPS, resulting in a mucoid phenotype. *Lon-1* or *lon-2* was inserted into the multiple cloning site of plasmid pBAD24, to produce plasmids pBAD*lon-1* and pBAD*lon-2*. Each plasmid was transformed into *lon*-deficient strain HDB98 (*lon510*), which contains a *cpsB::lacZ* fusion reporter [68]. Measurement of β-galactosidase activity allowed us to assess the ability of Lon-1 or Lon-2 to correct the defect in cpsB regulation by RscA. As shown in Figure 7A, after culture at 30°C in the presence of 0.1% arabinose, HDB98 containing pBAD*lon-1* (HDB98pBAD*lon-1*) exhibited β-galactosidase activity at levels similar to parental HDB98 and HDB98 containing vector alone (HDB98pBAD24). In HDB98pBAD*lon-2*, however, β-galactosidase activity levels were significantly reduced in comparison to HDB98, HDB98pBAD24, and HDB98pBAD*lon-1* (P<0.001). The reduction in β-galactosidase levels did not occur in the absence of arabinose (Figure 7B). Expression of soluble Lon-1 and Lon-2 by *E. coli* containing plasmids pBAD*lon-1* and pBAD*lon-2* was verified by western blot (Figure 7C, D, E).

**Figure 8:**
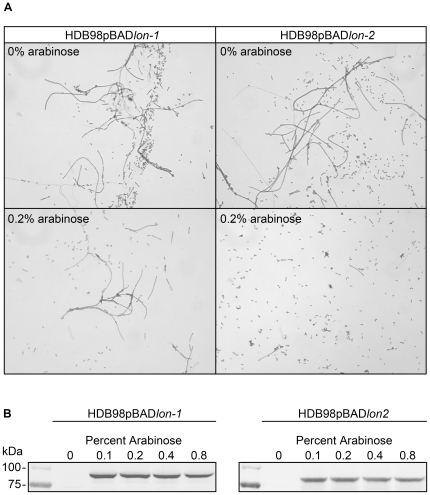
*lon-2*, but not *lon-1*, complements the filamentous phenotype of an *E. coli lon* mutant. (A) Brightfield microscopy of *E.coli* cultures grown at 37°C for 3.5 hours with or without 0.2% arabinose. Cells were applied to microslides, heat fixed, and visualized by Gram stain. (B) Western blot showing arabinose-induced Lon-1 and Lon-2 expression after culture at 37°C for 3.5 hours.

Another substrate for Lon is cell division inhibitor, SulA, which functions along with FtsZ protein to prevent disadvantageous cell division [39],[69]. Accumulation of SulA due to a Lon deficiency in *E. coli* causes impaired cell division resulting in a filamentous morphology, a phenotype exhibited in culture by *lon^510^* mutant HDB98 (data not shown). In the presence of 0.2% arabinose, HDB98pBAD*lon-1* produced long filamentous forms similar to the bacteria grown without arabinose and to HDB98, while in HDB98pBAD*lon-2*, arabinose induction of Lon-2 expression resulted in greatly reduced filament formation (Figure 8A). Expression of Lon-1 and Lon-2 was verified by western blot (Figure 8B). The ability of Lon-2 and not Lon-1 to complement HDB98 for filamentation and dysregulation of *cps* implies a differential capacity for these proteases to degrade RscA and SulA and supports a Lon-Ec-like role for Lon-2 in Borreliae.

We have shown that *B. burgdorferi* Lons-1 and -2 have characteristics in common with canonical Lon and with each other. Lon-1 protein displayed features of an ATP and Mg^2+^-dependent chaperone-protease, but *lon-1* did not complement an *E. coli lon* mutant, while *lon-2* corrected two distinct *lon* mutant phenotypes. Primary structural analysis showed that their respective ATPase and proteolytic domains are similar and characteristically Lon-like, but their substrate-binding domains are very different, suggesting that they may recognize divergent substrates. Taken together, this suggests that despite the structural similarities of Lon-1 and Lon-2, they appear to have distinct functional roles. The presence of Lon-1 as a unique feature of *Borrelia* would indicate that it has functions relevant to this genus as arthropod-transmitted pathogens. Specifically to *B. burgdorferi*, Lon-1 may function in host adaptation where it could be important as a protease during periods of rapid spirochete proliferation during a bloodmeal with its associated increases in protein biogenesis. It is also possible that during this period, Lon-1 could act as a chaperone for molecules that are not enzymatic substrates. Lon-2, on the other hand, functions as the canonical Lon, engaged in cellular homeostasis.

## Materials and Methods

### Ethics statement

All animal procedures were performed in compliance with the guidelines and with the approval of the Institutional Animal Care and Use Committee (IACUC) of Stony Brook University.

### Generation of polyclonal antisera to Lon-1 and Lon-2

For rabbit antiserum to Lon-1, New Zealand white rabbits were inoculated subcutaneously with 100 µg (400 µl) of recombinant Lon-1 in Freund's Complete Adjuvant. The rabbits were boosted three times over a period of a month (100 µg in 400 µl) in incomplete Freund's Incomplete Adjuvant followed by test a bleed and serological testing by ELISA and western blot to confirm reactivity to recombinant and native *B. burgdorferi* Lon-1. A final boost in PBS was given and the serum was collected four days later. For mouse antiserum to Lon-2, BALB/c mice were inoculated subcutaneously with 100 µg (200 µl) of recombinant Lon-2 in Complete Freund's Adjuvant. The mice were boosted subcutaneously (100 µg in 200 µl) two times with Freund's Incomplete Adjuvant followed by test a bleed and serological testing by ELISA and western blot to confirm reactivity to recombinant and native *B. burgdorferi* Lon-2. A final boost in PBS was given and the serum was collected three days later.

### Generation of recombinant proteins

Purified Lon-Ec was a gift of Dr A. Wali Karzai Department of Biochemistry and Cell Biology, Center for Infectious Diseases, Stony Brook University, Stony Brook, NY [42]. To generate recombinant Lon-1, the open reading frame of *lon*-1 (BB0253) was PCR amplified from B31MI [21] and ligated into the space between the NdeI and XhoI restriction sites of predigested vector pET30a(+) (Novagen, Gibbstown, NJ) using primers P1F and P2R (Table 2) to produce a construct that incorporated a 6-histidine tag at the C-terminal end. The resulting plasmid, pCK003.9 (Table 1), was introduced into BL21 Star™(DE3)pLysS (Invitrogen). *E. coli* were grown at 37°C in Tryptose Phosphate Glucose medium until an OD_600_ of ∼0.8 was reached, then induced at 37°C for 3–4 hours with 1.0 mM IPTG. Cell pellets were resuspended and sonicated for 30 seconds on ice in HisBind buffer (20 mM Tris HCl, pH 8.0, 0.5M NaCl, 20 mM imidazole) (Novagen) containing 0.2% NP40 (Pierce, Rockford, IL). Benzoase nuclease, 0.01%, (EMD Chemicals, Inc., Gibbstown, NJ) was added and the lysate was sonicated for an additional two minutes on ice. Clarified lysate was diluted in buffer A (20 mM Tris HCl, pH 8.0, 0.5M NaCl, 20 mM imidazole, 0.02% NP40) and applied to a column of HisTrap HP resin (GE Healthcare, Piscataway, NJ). Elution was carried out in three steps: 15%, 50%, and 100% of buffer B (20 mM Tris HCl pH 8.0, 0.5M NaCl, 1M imidazole, 0.02% NP40). The final buffer conditions were 20 mM Tris HCl pH8.0, 0.5M NaCl, 0.02% NP40, 0.5M imidazole, 10% glycerol. Exhaustive attempts to produce functional recombinant Lon-2 were unsuccessful. All constructs were confirmed by DNA sequencing. Recombinant protein expression and purification was done at the NBC Protein Expression Core, Wadsworth Center, Albany, NY.

### Site-directed mutagenesis of the Lon proteolytic domain

The QuicKChange II Site-Directed Mutagenesis Kit (Stratagene) and primers P3F and P4R (Table 2) were used to introduce a point mutation in the proteolytic domain of the lon-1 open reading frame, converting the catalytically active serine residue to alanine (S714A). The resulting construct in pET30a(+), pCK004.3 (Table 1) was confirmed by sequencing and used to transform expression host BL21 Star™(DE3)pLysS. Soluble mutant rLon-1 (rLon-1^S714A^) was expressed and purified as described above for wild-type rLon-1.

### *In vitro* proteolysis of *Borrelia*-SsrA tagged reporter protein

The purification of λ-CI-N reporter protein was performed as described by Choy et al [42] with some modifications. Briefly, plasmid pPW500 [70], which encodes the *λ-cI-N-trpAt* gene with no stop codon, was first modified by site directed mutagenesis. In this process, the *trpAt* terminator sequence of the reporter gene was replaced by the *B. burgdorferi ssrA*-tag sequence that was obtained from the tmRNA website (http://www.indiana.edu/~tmrna/). Thus, the final plasmid construct carried the *λ-cI-N* gene with the *B. burgdorferi ssrA*-tag sequence (*λ-cI-N*-*ssrA^Bb^*) and was transformed into an *E. coli clpP clpX lon* triple mutant. Bacterial cells were cultured in LB broth at 37°C and reporter expression was induced for 3 h with 1 mM IPTG when cultures reached mid-log phase. Finally, the λ-CI-N-SsrA^Bb^ reporter protein was purified by affinity chromatography with the aid of an internal His_6_ epitope as described [42]. The quality of the purified product was determined by SDS-PAGE followed by Coomassie blue staining, and the size of each protein band was verified by MALDI-TOF mass spectroscopy. *In vitro* proteolysis assays were carried out with a final concentration of 1.5 µM of either rLon-1 or rLon-Ec, 10 µM λ-CI-N-SsrA^Bb^ or 0.3 mg/ml insulin as substrates, an ATP regeneration system (50 mM creatine phosphate, 80 µg/ml creatine kinase [56], 4 mM ATP), and a minimal activity buffer (50 mM Tris-HCl, pH 8.0, 10 mM MgCl_2_, 1 mM dithiothreitol). An additional 20 mM of dithiothreitol was included in assays with insulin. The reaction mixtures were incubated at 30°C and a fraction of each sample was collected at various time points for analysis by 15% Tris-tricine SDS-PAGE. Protein bands were quantified by using an Odyssey infrared imaging system (LiCor Biosciences, Lincoln NE).

### Phylogenetic amino acid sequence analysis

Sequences and their annotations were obtained from the Swiss-Prot Protein knowledgebase. The Clustal W algorithm [71] was used for sequence alignments and Mega 3 software version 3.1 [72] for phylogenetic analyses. Neighbor joining methods [73] were used to build phylogenetic trees. Percentage support values were obtained through a bootstrap procedure. Analyses were also carried out using nucleotide sequences.

### Complementation of *E. coli* lon mutant HDB98

Wild-type *E. coli* HDB97 and *lon* mutant HDB98 (*lon*510) were the gift of Dr. Harris D. Bernstein, Genetics and Biochemistry Branch, National Institute of Diabetes and Digestive and Kidney Diseases, National Institutes of Health. *B. burgdorferi lon-1* was amplified from *B. burgdorferi* strain B31A3 [74] by PCR using primers P50F and P51R and cloned into the NcoI restriction site of plasmid pBAD24 [75] which confers ampicillin resistance (American Type Culture Collection, Manassas, VA). The insert was placed eight nucleotides downstream from the optimized ribosomal binding site to form plasmid pBAD*lon-1*. *Lon-2* was amplified by PCR using primers P52R F and P53R and cloned into a region between the NheI and KpnI restriction sites of pBAD24, also leaving eight nucleotides between the pBAD24 ribosomal binding site and the *lon-2* ATG start site to yield plasmid pBAD*lon-2*. HDB98 was subsequently transformed with pBAD*lon-1* and pBAD*lon-2* to form strains HDB98pBAD*lon-1* and HDB98pBAD*lon-2*. Both constructs were confirmed by nucleotide sequencing.

### β-galactosidase assay

Measurement of β-galactosidase activity of *E. coli* strains was done as previously described [76],[77]. HDB97 and HDB98, which contain a *cpsB::lacZ* reporter fusion, were cultured in LB medium. HDB98pBAD24 (containing empty pBAD24 vector), HDB98pBADlon-1 and HDBpBADlon-2 were cultured in LB medium with ampicillin and 0.1% L-arabinose to induce expression of *B. burgdorferi* Lons. Activity units were calculated as follows:
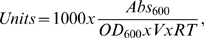
where *OD_600_C* represents the optical density of the culture, *V*, the culture volume analyzed (ml), *RT*, the reaction time (min), and *Abs_420_*, the absorbance reading after color development. Each strain was analyzed in triplicate and the experiment was repeated a total of five times. Data was tested for statistical relevance using InStat 3.0 Software (GraphPad Software, Inc., San Diego, CA).

### Assays of enzymatic activity

Hydrolysis of casein by *B. burgdorferi* rLon-1 was used as a measure of proteolytic activity and was based on the method of Twining et al. [78]. Preparations of rLon-1 and rLon-1^S714A^ contained 20 mM Tris-HCl, 0.5M NaCl, 0.5M imidazole, 0.02% NP40, and 10% glycerol. To assay for proteolytic activity, 5 µg of enzyme was incubated in 1.5 ml microcentrifuge tubes for 3 hours at 37°C with 100 µg of FITC-labeled casein, type I (Sigma). Final reaction conditions after addition of Lon proteins were: 50 mM Tris-HCl, pH 8, 10 mM MgCl_2_, 4 mM ATP, 25 mM NaCl, 25 mM imidazole, 0.001% NP40, and 0.5% glycerol in a total volume of 100 µl. In assays designed to test for the influence of Mg^2+^ on rLon-1 proteolytic activity, 10 mM MgCl_2_ was omitted from the reaction buffer. Proteolysis was terminated by the addition of 10 µl of bovine serum albumin (10 mg/ml stock solution) immediately followed by 100 µl of 10% trichloroacetic acid (TCA), and 10 minutes of incubation on ice. The reaction contents were then centrifuged at 16,000× g to separate TCA insoluble material. For each sample replicate, 125 µl of the TCA soluble supernatant fraction was transferred to the well of a 96-well plate (Falcon - Becton Dickenson) and neutralized by the addition of 125 µl of 0.5 M CHES-Na, pH 12. Fluorescence was measured in a SpectraMax M2 microplate reader (Molecular Devices, Sunnyvale, CA) at wavelengths of 490 nm (excitation) and 525 nm (emission). The assay for measurement of optimum pH for casein degradation was carried out at 26°C for 16 hours and the following buffers were used to achieve the desired assay pH: 50 mM MES (pH 6.0, 6.5, 7.0), 50 mM Tris-HCl (pH 7.5, 8.0, 8.5), 50 mM 2-aminomethanepropanediol-HCl (AMPD) (pH 9.0, 9.5, 10.0), 50 mM CAPS (pH 10.5) [52].

The ATPase activity of rLon-1 and rLon-1^S714A^ was measured colorimetrically by use of a kit-based assay which utilized the dye malachite green to detect the liberation of inorganic phosphate resulting from enzymatic ATP hydrolysis (Innova Biosciences, Cambridge, UK). The manufacturer's protocol was followed for all assays and utilized 5 µg of enzyme per assay reaction. Components of the enzyme purification buffer resulted in final concentrations of 12.5 mM NaCl, 12.5 mM imidazole, 0.0005% NP40, and 0.25% glycerol in the reaction. Absorbances were measured in a SpectraMax M2 microplate reader at a wavelength of 635 nm.

### Assay of chaperone-like activity

Reduction of its disulfide bonds induces the separation of insulin A and B chains with the attendant aggregation of the B chain [63]. The assay for chaperone-like activity is based upon the protection of reduced B-chain from aggregation by an added chaperone-like molecule. The degree of protection conferred is monitored by the measurement of resultant light scattering. In our assay, a modification of Farahbakhsh et al [79], 15 µg of bovine insulin was added to 25 µg of wild-type rLon-1 or mutant rLon-1^S714A^ protein in the presence of 10 mM sodium phosphate, pH 7.2, 20 mM dithiothreitol, 500 mM NaCl, 50 mM imidazole, 1% glycerol, 20 mM MgCl_2_, and either 4 mM or 10 mM ATP (total volume of 500 µl) in a 1-cm plastic cuvette. Light scattering was measured over time at 20°C in an Ultrospec 4000 UV/Vis spectrophotometer (Pharmacia Biotech, Piscataway, NJ) at a wavelength of 360 nm. A water-jacketed cell changer in conjunction with a heater/circulator (Neslab Instruments, Newington, NH) was used to ensure that the temperature was maintained. The data were collected and analyzed by SWIFT software in the kinetics mode (Pharmacia Biotech). Wild-type rLon-1 and mutant rLon-1^S714A^ were also analyzed for their ability to degrade reduced insulin under chaperone assay conditions. These digestions were carried out in 1.5 ml microcentrifuge tubes under the conditions described for the chaperone assay, with the exceptions that 150 µg of insulin was used. Incubation was done for 2 hours at 37°C.

### SDS-PAGE and western blotting

Tris-glycine SDS-PAGE of recombinant *B. burgdorferi* Lon proteins (1 µg) was conducted as described previously [80] in gels of 12.5% polyacrylamide. For *E. coli* used in complementation experiments, 1 ml of culture was centrifuged and resuspended in 100 µl 1× SDS-PAGE sample buffer, and boiled. Samples (10 µl) were electrophoresed by 12.5% Tris-glycine SDS-PAGE. In all cases, electrophoretic transfer of protein was carried out over a period of 16 hours in 0.01M CAPS buffer, pH 11. For analysis of Lon-1 insulin degradation in the chaperone assay, 100 µl of reaction mixture was added to 50 µl of 3× SDS-PAGE sample buffer and boiled. For each condition, 10 µl was electrophoresed by Tris-tricine SDS-PAGE using a 16.5% separating gel, a 10% spacer gel, and a 4% stacking gel. Equivalent volumes of degraded tagged reporter protein (2.2 µg) and insulin (4.5 µg) from SsrA experiments were analyzed using a 15% Tris-tricine separating gel alone. For direct staining, the gels were fixed in 50% methanol-12% acetic acid then stained with 0.1% Coomassie blue R-250 in 50% methanol-12% acetic acid. For western blotting, Lon-1 and Lon-2 in lysates were detected using rabbit and mouse polyclonal antiserum, respectively. Infrared-conjugated secondary antibodies were goat anti-rabbit IgG (H+L) and goat anti-mouse IgG (H+L) (Rockland, Gilbertsville, PA). Glyceraldehyde-3-phosphate dehydrogenase (GAPDH) was detected using goat polyclonal antibody (GenScript Corp., Piscataway, NJ) and donkey anti-goat IgG (H+L) secondary antibody. Bands were visualized by use of an infrared scanner (Odyssey Infrafred Imaging System, LiCor Biosciences).

### Accession/ID numbers for proteins sequences mentioned in the text

Swiss-Prot Protein knowledgebase: *Borrelia burgdorferi* Lon-1 (Q59185), *Borrelia burgdorferi* Lon-2 (O51558), *Borrelia recurrentis* Lon-1 (B5RR69), *Borrelia recurrentis* Lon-2 (B5RPV9), *Borrelia duttoni* Lon-1 (B5RL78), *Borrelia duttoni* Lon-2 (B5RMG3), *Borrelia hermsii* Lon-1 (B2RZW3), *Borrelia hermsii* Lon-2 (B2S0W0), *Borrelia garinii* Lon-1 (Q662B2), *Borrelia garinii* Lon-2 (Q660R0), *Borrelia afzelii* Lon-1 (Q0SNQ8), *Borrelia afzelii* Lon-2 (Q0SMP4), *Treponema pallidum* Lon (O83536), *Treponema denticola* Lon (Q73PX5), *Leptospira biflexa* (B0SLS1), *Leptospira borgpetersenii* (Q04VA7), *Leptospira interrogans* (Q72UP9), *Escherichia coli* Lon (P0A9M0), *Bacillus subtilis* Lon (P37945).
